# Metabolic rewiring in fat-depleted Drosophila reveals triglyceride:glycogen crosstalk and identifies cDIP as a new regulator of energy metabolism

**DOI:** 10.21203/rs.3.rs-4505077/v1

**Published:** 2024-10-18

**Authors:** W. Mike Henne, Rupali Ugrankar-Banerjee, Son Tran, Jade Bowerman, Blessy Paul, Lauren Zacharias, Thomas Mathews, Ralph DeBerardinis

**Affiliations:** UT Southwestern Medical Center; UT Southwestern Medical Center; UT Southwestern Medical Center; UT Southwestern Medical Center; University of Texas Southwestern; UT Southwestern; University of Texas Southwestern Medical Center; Howard Hughes Medical Institute

**Keywords:** Fatty acid synthase (FASN), glycolysis, glycogen, triglyceride (TG), nicotinamide adenine dinucleotide phosphate (NADPH)

## Abstract

Tissues store excess nutrients as triglyceride or glycogen, but how these reserves are sensed and communicate remains poorly understood. Here we identify molecular players orchestrating this metabolic balance during fat depletion. We show fat body (FB)-specific depletion of fatty acyl-CoA synthase FASN1 in Drosophila causes near-complete fat loss and metabolic remodeling that dramatically elevates glycogen storage and carbohydrate metabolism. Proteomics and metabolomics identify key factors necessary for rewiring including glycolysis enzymes and target-of-brain-insulin (tobi). FASN1-deficient flies are viable but starvation sensitive, oxidatively stressed, and infertile. We also identify CG10824/cDIP as upregulated in FASN1-depleted Drosophila. cDIP is a leucine-rich-repeat protein with homology to secreted adipokines that fine-tune energy signaling, and is required for fly development in the absence of FASN1. Collectively, we show fat-depleted Drosophila rewire their metabolism to complete development, and identify cDIP as a putative new cytokine that signals fat insufficiency and may regulate energy homeostasis.

## Introduction

Organisms must sense and allocate nutrients to metabolism, development, and reproduction to maintain homeostasis and populations^1,2^. Dietary carbohydrates are mobilized to glucose and catabolized through glycolysis, the tricarboxylic acid (TCA) cycle, and oxidative phosphorylation to yield carbon dioxide and ATP, the cellular energy currency^3–5^. Excess glucose is utilized for the *de novo* synthesis of fatty acids and triglycerides (TG) and incorporated into lipid droplets (LD) in the adipose tissue, or condensed to glycogen, a polymeric form of glucose stored preferentially in liver and muscle^3,4,6–8^. Perturbations in energy storage or utilization are linked to metabolic disorders such as obesity, Type 2 diabetes, cardiovascular disease, and cancers^4,5,9–11^. However, the metabolic decision-making that dictates how tissues invest in and balance fat and glycogen storage, and how these energetic reserves are sensed and communicate to ensure homeostasis and development, are not well understood^1^.

*Drosophila melanogaster* is a powerful model to interrogate metabolic balance and human metabolic diseases. Not only does *Drosophila* carry functional homologs to ~75% of human disease genes, but key metabolic pathways such as insulin and TOR signaling are evolutionarily conserved in flies^10,12,13^. Like in humans, *Drosophila* can become obese, lipodystrophic, or diabetic in response to caloric cues or genetic mutations^14–16^. The primary nutrient storage tissue in the fly is the fat body (FB) which shares features of the mammalian adipose tissue and liver^17,18^. The embryonic FB is comprised of a continuous monolayer of ~2200 polygonal cells whose numbers remain unchanged but increase in size during larval development^19–22^. In feeding larvae, most dietary carbohydrates are converted to TG and assimilated into LDs within the FB. Glycogen is also detected in the FB, albeit at significantly lower concentrations than lipids. Critically, the role of glycogen in fly adipose tissue is not clear, however recent studies indicate that glycogen may be important for maintaining hemolymph trehalose, the predominant circulating sugar in *Drosophila*, present at ~100-times the level of glucose^12,16,23^.

Commitment to pupariation and metamorphosis requires that feeding larvae reach a biomass checkpoint called ‘critical weight’. Much of this body mass is contributed by TG-rich LDs in the FB^24–26^. During metamorphosis juvenile larvae are transformed into adult flies. This non-feeding developmental stage is energetically expensive and requires large-scale tissue remodeling, imaginal cell differentiation, and growth^27–29^. However, there is no consensus as to whether FB TG is necessary for pupal development. For example, lipodystrophic *Drosophila* models encoding mutations in fly homologs of *Lipin*, a phosphatidic phosphatase for making the TG precursor DAG, and *Seipin*, required for normal LD biogenesis, still achieve critical mass despite having smaller adipose tissue size and/or greatly reduced TG content. In fact, ~60% of *dLipin* mutants survive to the late pharate adult stage, and ~40% became adults. Similarly, no lethality is observed for *dSeipin* mutants and adults were viable and fertile^15,30,31^. Recent studies instead point to the blood-circulating sugar trehalose as an essential energy source for metamorphosis^25,32,33^. These observations suggest FB TG stores may not be critical for fly development, but how they specifically contribute to metamorphosis and development, and whether other reserves provide additional energy or biomass remains poorly understood^25,32,33^.

TG in the FB is produced from two lipid pools: absorbed lipids from the blood by lipophorin trafficking or via *de novo* lipogenesis that requires fatty acid synthase (FASN)^9,34^. FASN is evolutionarily conserved and catalyzes the synthesis of long chain fatty acids from carbohydrate-derived acetyl-CoA, malonyl CoA, and the electron donor nicotinamide adenine dinucleotide phosphate (NADPH)^11,35^. *Drosophila* encode three *FASN* genes with distinct tissue expressions. *FASN1/CG3523* is ubiquitously expressed with highest expression in the FB, whereas *FASN2/CG3524* and *FASN3/CG17374* are expressed primarily in the body wall, oenocytes, and hemocytes (FASN2), or oenocytes and reproductive system (FASN3)^4,28,36,37^. *Drosophila* hypomorphs for *FASN1* and *FASN2* exhibit reduced larval and adult TG, while null mutants of *FASN1* and *FASN3* display embryonic or early larval developmental arrest^9,28^. Fatty acid synthesis is thus crucial for the generation of energy reserves, cellular membranes, and protection from sugar toxicity^4,35^. However, how FASN-driven lipid synthesis is coordinated with carbohydrate metabolism to enable homeostasis and development remains incompletely understood.

Here we identify molecular players that orchestrate the metabolic balance between lipid and carbohydrate utilization, and dissect how TG and glycogen stores contribute to *Drosophila* development. We find that most TG in the larval FB is generated via FASN1-mediated de novo lipogenesis, but surprisingly both FASN1 and its TG products in the FB are dispensable for larval development and metamorphosis. FB-specific *FASN1R*NAi knockdown (defined here as *FASN1*^*FB-RNAi*^) leads to fully developed larvae that lack >85% of TG lipid biomass, but remarkably still complete their developmental cycle. We find this is achieved through metabolic re-wiring that drastically elevates FB glycogen storage (with an excess of ~20-fold increase in glycogen compared to controls) and carbohydrate metabolism through glycolysis and trehalose export. *FASN1*^*FB-RNAi*^ adults are viable but lack detectable TG stores, and females are infertile due to defects in ovarian differentiation and oocyte development. FASN1-depleted adults are also sensitive to nutrient deprivation, exhibit shortened median lifespan, and larvae display signatures of redox imbalance including lipid peroxidation. Through proteomics we uncover a putative secreted cytokine, Common Dpr-interacting protein (cDIP)/CG10824 that is required for larval development when fat is depleted. cDIP is highly upregulated in *FASN1*^*FB-RNAi*^ FBs and shows homology to secreted leucine-rich repeat proteins that regulate vertebrate insulin/IGF-like signaling. In line with this, we find cDIP FB-specific knockdown elevates fat storage, and is required for development when fat body lipid stores are depleted. Collectively, we hypothesize that *FASN1*^*FB-RNAi*^ Drosophila signal fat deficiency by secreting cDIP to coordinate fat-to-carbohydrate metabolic homeostasis, ensuring metabolic adaptation and animal development.

## Results

### Fat body specific-loss of FASN1 leads to triglyceride-depleted but viable *Drosophila*

**Figure 1:**
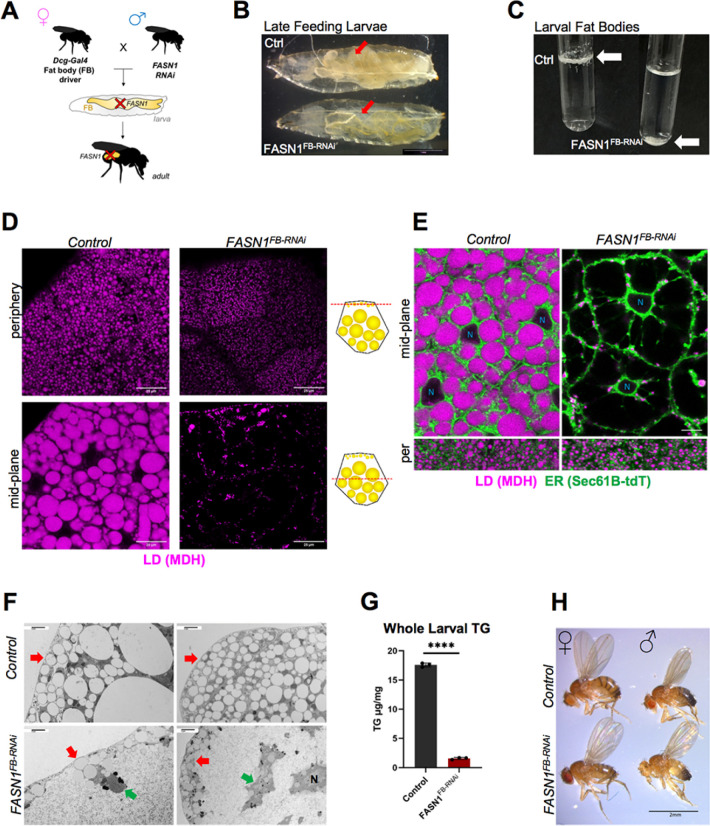
Loss of FASN1 in *Drosophila* fat body produces virtually fat less flies A) Cartoon of RNA interference-mediated knockdown using the UAS/Gal4 system; parental cross between females carrying tissue (fat body)-specific Dcg-Gal4 driver and males containing the *UAS-FASN1*^*RNAi*^ transgene generate progeny lacking *FASN1* in the FB. B) Control (*Dcg-Gal4*) and *FASN1*^*FB-RNAi*^ (*Dcg-Gal4>UAS-FASN1*^*RNAi*^) age-matched late feeding/pre-wandering third instar larvae (L3). Scale bar 1 mm. C) Buoyancy-based test of fat content in extracted FBs from control and *FASN1*^*FB-RNAi*^ larvae. D) Confocal images of control and *FASN1*^*FB-RNAi*^ larval (L3) FB periphery and mid-plane sections stained with LD stain monodansylpentane (MDH). Scale bar 25 μm. E) Confocal images of control and *FASN1*^*FB-RNAi*^ FBs at their mid-plane and periphery from larvae expressing ER marker (*Dcg-Gal4>UAS-Sec61B-tDTomato*) and stained with MDH. *FASN1* loss results in adipocytes empty of LDs. F) TEM micrographs of control and *FASN1*^*FB-RNAi*^ FB cells showing peripheral LDs (pLDs, red arrows). *FASN1*^*FB-RNAi*^ cell appears vacant of medial LDs (mLDs) and contains little ‘islands’ of ER, LDs, and other organelles (green arrows). G) Total triglycerides (TG) measured by thin layer chromatography (TLC) in whole L3 pre-wandering control and *FASN1*^*FB-RNAi*^ larvae. H) Size comparison of control and *FASN1*^*FB-RNAi*^ female (left) and male (right) adult flies. Scale bar 2 mm.

The fat body (FB) is the central metabolic organ in *Drosophila*, and represents much of the larval biomass necessary to achieve the critical weight for metamorphosis^18,24–26^. Most of the FB lipid mass is composed of lipid droplet (LD) triglycerides (TGs) produced from *de novo* lipogenesis in the FB^9,34,36^. To genetically manipulate these fat stores, we utilized the FB tissue-specific driver *Dcg-Gal4*^16,38^ to selectively RNAi-deplete the major *de novo* fatty acyl-CoA synthase *FASN1*, which we previously identified as the major FASN isoform in FB TG synthesis^34^ (Fig 1A). FB-specific *FASN1* knockdown (*Dcg-Gal4>FASN1*^*RNAi*^, denoted *FASN1*^*FB-RNAi*^) led to visible reduction of larval fat deposits, still *FASN1*^*FB-RNAi*^ larvae developed in tandem with age-matched controls (*Dcg-Gal4*) and grew to full size. However, the adipose tissue in L3 pre-wandering larvae (~100 to 108 h AEL, After Egg Laying) appeared translucent instead of the opaque white color typical of fat-engorged tissue (Fig 1B, **red arrows**). In line with this, isolated FBs from *FASN1*^*FB-RNAi*^ larvae now sank in PBS buffer, indicating they lost the buoyancy provided by their fat stores (Fig 1C, **white arrows**). Of note, the amount of FB tissue itself was not reduced and contained normal sized adipocytes, despite general loss of fat content, indicating tissue development was not altered by FASN1 loss (**SFig 1A,B**). Perturbations in FB fat storage is known to cause ectopic accumulation of lipids in other tissues such as the mid-gut^39^, however, gut TG levels were significantly reduced in *FASN1*^*FB-RNAi*^ larvae relative to controls (**SFig 1C**).

To determine how FASN1 depletion impacted LDs in the FB, we conducted z-section confocal imaging. *FASN1*^*FB-RNAi*^ larvae displayed a near complete lack of large LDs in the tissue mid-plane (mid-plane LDs, mLDs) within FB cells. However, *FASN1*^*FB-RNAi*^ larvae still exhibited small peripheral LDs (pLDs) near the tissue edges exposed to the animal hemolymph (Fig 1D,E). This is consistent with previous work indicating these pLDs are not dependent on *de novo* lipogenesis within the FB, but rather potentially derived from dietary lipids via lipophorin-mediated lipid trafficking from other tissues^34^. pLDs near the cell surface were also observed by transmission electron microscopy (TEM), whereas most of the *FASN1*^*FB-RNAi*^ FB cell cytoplasm looked vacant, except for small “islands” of organelles (Fig 1F). As expected, total larval TG was significantly impacted as FB-specific FASN1-depletion led to loss of ≥ 85% larval TGs compared to control larvae (Fig 1G). To determine whether LD depletion in *FASN1*^*FB-RNAi*^ FB cells occurred in a cell autonomous manner, we also knocked down *FASN1* using the ubiquitous but mosaic driver *da-Gal4*, which selectively induces RNAi in a subset of FB cells. Indeed, *da-Gal4>FASN1*^*RNAi*^ larval FB tissues exhibited a mosaic pattern of LD-depleted FB cells (also positive for PM marker mcd8-GFP) adjacent to unaffected cells (no mcd8-GFP) with typical intracellular LD populations (**SFig 1D**). Importantly, LD-depleted cells still contained pLDs near the tissue surface, but lacked the larger mLDs near the tissue mid-plane, consistent with FASN1 loss primarily impacting the large LD population. Collectively, this indicates that FASN1 loss in FB cells causes cell autonomous blockage of *de novo* lipogenesis and abolishes TG storage in mLDs.

Despite their dramatic reduction of TG, FASN1^FB-RNAi^ larvae surprisingly pupated with no notable developmental delay, completing metamorphosis and emerging as normal sized and apparently healthy flies (Fig 1H). Collectively, this indicates that FASN1 is not necessary in the FB for animal development, and that *Drosophila* larvae can reach critical weight and successfully complete development despite the loss of >85% of their FB TG stores.

### FASN1 is the primary FASN isoform for TG storage in the FB

**Figure 2:**
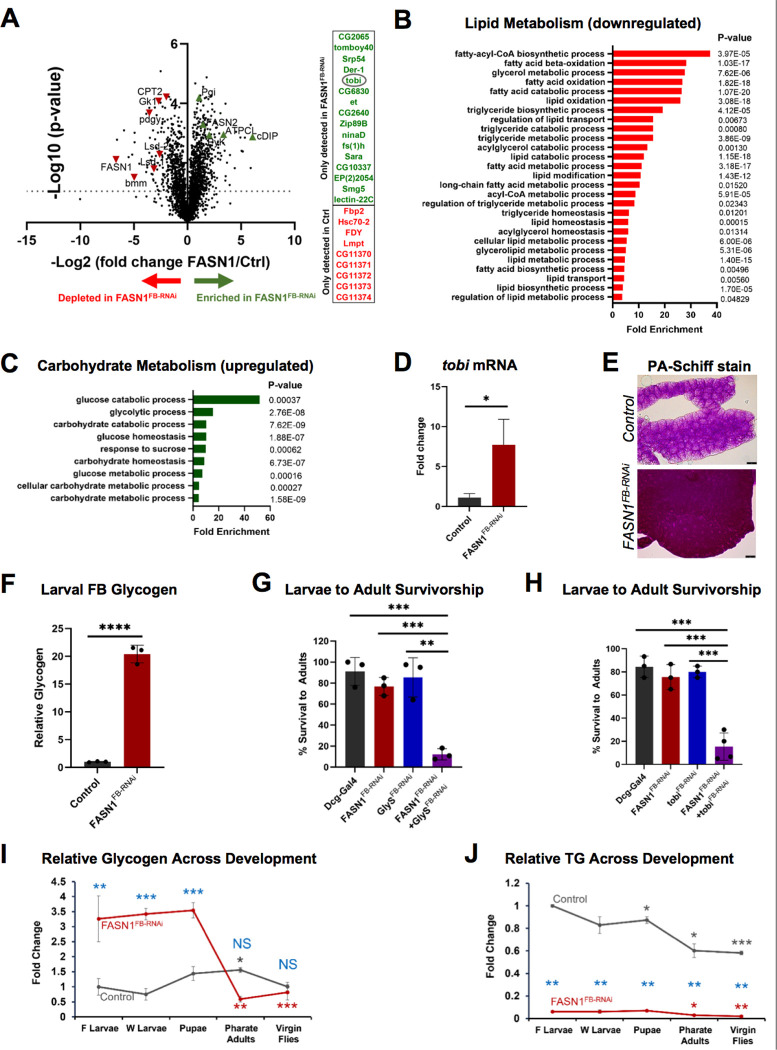
*FASN1*-deficient larvae channel dietary glucose to glycogen as energy reserves A) Volcano plot of proteins detected by LC-MS/MS analyses performed in FBs isolated from control and *FASN1*^*FB-RNAi*^ L3 larvae. B) Lipid metabolism processes were revealed to be amongst the most downregulated in *FASN1*^*FB-RNAi*^ FBs. GO enrichment analysis of proteomics data was performed using Pangea. C) Carbohydrates metabolism processes downregulated in *FASN1*^*FB-RNAi*^ FBs (Pangea) D) Relative expression of tobi mRNA in isolated FBs from control and *FASN1*^*FB-RNAi*^ larvae, measured by QPCR. E) Periodic acid-Schiff (PAS) glycogen stain of larval FBs from control and *FASN1*^*FB-RNAi*^ larvae. F) Quantification of glycogen extracted from control and *FASN1*^*FB-RNAi*^ larval FBs. G) Percentage of L3 larvae (control, *FASN1*^*FB-RNAi*^, *GlyS*^*FB-RNAi*^, *FASN1*^*FB-RNA i*^+ *GlyS*^*FB-RNAi*^) successfully completing metamorphosis to yield adult flies. H) Percentage of L3 larvae (control, *FASN1*^*FB-RNAi*^, *tobi*^*FB-RNAi*^, *FASN1*^*FB-RNA i*^+ *tobi*^*FB-RNAi*^) successfully completing metamorphosis to adult flies. I) Relative glycogen levels measured in control and *FASN1*^*FB-RNAi*^ animals during distinct developmental stages: Feeding Larvae: F Larvae (~100–108 h AEL), Wandering Larvae: W Larvae (non-feeding, ~108–120 h AEL), Pupae (~132–140 h AEL), Pharate Adults (~205–215 AEL), Virgin Flies (male + female, 0 to 2 h post-eclosion). AEL = After Egg Laying. *P<0.05: FASN1 FL, WL, P, PA, VF relative to Dcg FL, *P<0.05: Dcg WL, P, PA, VF relative to Dcg FL, *P<0.05 FASN1 WL, P, PA, VF relative to FASN1 FL. J) Relative triglyceride levels measured in control and *FASN1*^*FB-RNAi*^ animals during distinct developmental stages: Feeding Larvae: F Larvae (~100–108 h AEL), Wandering Larvae: W Larvae (non-feeding, ~108–120 h AEL), Pupae (~132–140 h AEL), Pharate Adults (~205–215 AEL), Virgin Flies (male + female, 0 to 2 h post-eclosion). AEL = After Egg Laying. *P<0.05: FASN1 FL, WL, P, PA, VF relative to Dcg FL, *P<0.05: Dcg WL, P, PA, VF relative to Dcg FL, *P<0.05 FASN1 WL, P, PA, VF relative to FASN1 FL.

*Drosophila* FB lipids are proposed to play important roles in animal development including as an energy source for metamorphosis, biomaterials for tissue development, as well as a metabolic buffer to counter caloric overload^10,26,28^. Since TG-depleted *FASN1*^*FB-RNAi*^ FBs still supported fly development, we investigated what factors and pathways were necessary for metamorphosis into adults in the absence of FASN1 in the FB. We conducted unbiased global proteomics of isolated FBs from control and *FASN1*^*FB-RNAi*^ pre-wandering L3 larva using liquid-chromatography coupled with tandem mass spectrometry (LC-MS/MS). Principal component analysis (PCA) revealed control and *FASN1*^*FB-RNAi*^ FBs exhibited significant differences in the relative abundances of many proteins (**SFig 2A**). Proteomics also confirmed efficient knockdown of FASN1 as it was among the most decreased of all detected proteins in its abundance in *FASN1*^*FB-RNAi*^ larval FBs (Fig 2A). Similarly, we noted significant decreases in the abundance of other proteins important for TG synthesis or storage, such as the glycerol kinase Gk1 (important for making glycerol-3-phosphate and TG), and the perilipins Lsd1 and Lsd2, as well as proteins involved in TG breakdown such as Bmm (mammalian ATGL homolog), and pdgy and CPT2 (involved in fatty acid β-oxidation) (Fig 2A). GO term analysis revealed that lipid metabolic processes, particularly those related to fatty acid and TG biosynthesis, as well as fatty acid catabolism and oxidation were among the most significantly decreased pathways in *FASN1*^*FB-RNAi*^ FBs (Fig 2B). Notably, FASN2 protein levels were moderately increased in *FASN1*^*FB-RNAi*^ FBs (~3-fold), but FASN3 was undetected in both control and *FASN1*^*FB-RNAi*^ FBs likely due to low expression in this tissue (Fig 2A). Quantitative mRNA measurements by qPCR of extracted FBs further confirmed successful *FASN1* targeting by RNAi, and mild upregulation of *FASN2* transcripts in FASN1-depleted fat tissue (**SFig 2B**). This suggests that in the absence of FASN1, the FB still expresses FASN2 and can produce fatty acyl-CoAs, likely to supply lipids for membrane and organelle biosynthesis. In line with this, GO analysis indicated that phospholipid biosynthesis and metabolism pathways were upregulated in *FASN1*^*FB-RNAi*^ FBs relative to controls (**SFig 2C**).

To determine whether loss of FASN2 or FASN3 would similarly deplete TG FB stores like FASN1 loss, we knocked down *FASN3* in the FB using a *UAS-RNAi TRiP* line. Since a *UAS-RNAi* line for *FASN2* was unavailable, we instead examined the FB of an established *FASN2* mutant. Indeed, global loss FASN2 only mildly altered LD storage in the FB relative to *FASN1*^*FB-RNAi*^, while *FASN3*^*FB-RNAi*^ had no obvious LD defects in the FB and resembled control tissue (**SFig 2D**). This implies that FASN1 may generate fatty acyl CoAs that are channeled primarily into LD-TG in the larval FB.

### FASN1-depleted FBs exhibit elevated glycogen storage and require target-of-brain-insulin (tobi) for development

FB proteomics revealed that many proteins were significantly elevated in *FASN1*^*FB-RNAi*^ compared to controls, particularly those involved in carbohydrate metabolism. GO term analysis indicated that carbohydrate catabolic processes, and glycolytic processes were among the top scoring pathways increased in *FASN1*^*FB-RNAi*^FBs (Fig 2C). Among these up-regulated carbohydrate-related proteins was target-of-brain-insulin (tobi), which was only detected in the proteomics of *FASN1*^*FB-RNAi*^ FBs (Fig 2A), and whose mRNA level increased ~8-fold in *FASN1*^*FB-RNAi*^ FBs compared to controls (Fig 2D). The *tobi* gene encodes a conserved alpha-glucosidase expressed primarily in FB and gut, and is activated during sugar mobilization in the FB^40,41^. Since alpha-glucosidases mediate glycogen breakdown to glucose, we examined how FB glycogen levels compared between control and *FASN1*^*FB-RNAi*^ FBs. Strikingly, periodic acid-Schiff (PAS) staining revealed significantly concentrated glycogen deposits in *FASN1*^*FB-RNAi*^ FBs compared to controls, and biochemical measurements confirmed a remarkable >20-fold glycogen increase in *FASN1*^*FB-RNAi*^ FBs (Fig 2E,F). Increased glycogen granules were also detected in *FASN1*^*FB-RNAi*^ FB by imaging *Glycogenin-YFP*, as glycogenin functions as a primer for glycogen polymerization^42^(**SFig 2E**). QPCR analyses showed elevated expression of other key enzymes involved in glycogen synthesis (glycogenesis) and glycogen breakdown (glycogenolysis) (**SFig 2F**). This suggested that, in the absence of TG stores, larvae may shunt dietary nutrients into glycogen for energy storage and utilization in the FB. From this, we hypothesized that *FASN1*^*FB-RNAi*^ larvae relied on carbohydrate metabolism for development.

To test this, we RNAi depleted the glycogen synthesis enzyme GlyS, or the glycogen mobilization enzyme tobi, either alone or in combination with FASN1 specifically in the larval FB. Whereas RNAi loss of GlyS or tobi on their own did not impact larval development into flies, depletion of either in combination with *FASN1* ^*FB-RNAi*^ led to developmental arrest during the pupal stage (Fig 2G,H). Next, we determined whether FASN1-depleted larvae utilized glycogen during pupal development to complete metamorphosis. We monitored TG and glycogen levels through distinct developmental stages of larval growth, pupation, and fly emergence. Indeed, we observed that during the transition from the pupal to pharate adult (late pupal) stage, glycogen levels in *FASN1*^*FB-RNAi*^ animals (but not controls) declined rapidly (Fig 2I). In fact, newly hatched *FASN1*^*FB-RNAi*^ adults contained less than 10% of their initial glycogen stores, consistent with glycogen being heavily utilized to support fly development. In contrast, glycogen amounts in controls remained steady through metamorphosis and into adulthood (Fig 2I). In contrast, control animals appeared to mobilize FB TG stores during development, particularly during the pupal period when major tissue remodeling and pharate adult development occurs^25^ (Fig 2J). A small decrease in TG levels of *FASN1*^*FB-RNAi*^was also observed, however, the energy available through TG lipolysis in this fat-deficient animal is likely negligible (Fig 2J). Collectively, this indicates that TG-depleted *FASN1*^*FB-RNAi*^ larvae exhibit significantly elevated carbohydrate storage in the form of FB glycogen, and functionally require glycogen synthesis and breakdown for development into flies.

### *FASN1*^*FB-RNAi*^ larvae require glycolysis for development

**Figure 3:**
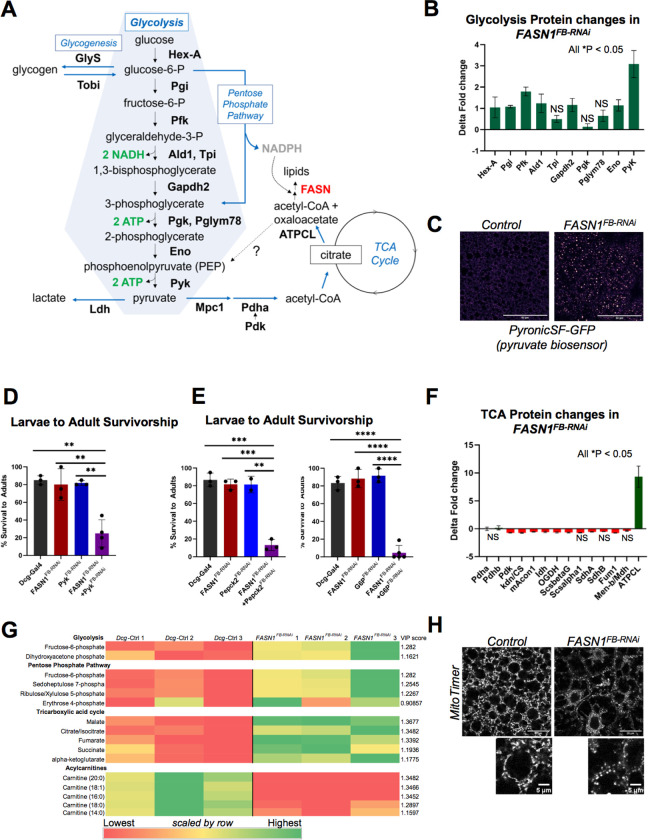
Larvae lacking FASN1 elevate glycolysis and PPP while down-regulating mitochondrial TCA and ETC A) Schematic representing the fate of glucose and role of FASN in *de novo* lipogenesis. B) Relative increases (delta fold) in protein levels of glycolysis enzymes in *FASN1*^*FB-RNAi*^ FBs revealed by proteomics profiling. C) Expression of fluorescent pyruvate biosensor UAS-PyronicSF-GFP in control and *FASN1*^*FB-RNAi*^ in larval FBs: Fire-LUT pseudo-colored (Fiji). Scale Bar 50 μm. D) Percentage of L3 larvae (control, *FASN1*^*FB-RNAi*^, *Pyk*^*FB-RNAi*^, *FASN1*^*FB-RNAi*^+ *Pyk*^*FB-RNAi*^) successfully completing metamorphosis to adult flies. E) Percentage of control and *FASN1*^*FB-RNAi*^ larvae, and *G6P*^*FB-RNAi*^ or *Pepck2*^*FB-RNAi*^ larvae with or without *FASN1* successfully completing metamorphosis to adult flies. F) Relative changes (delta fold) in tricarboxylic acid cycle (TCA) enzymes in *FASN1*^*FB-RNAi*^ FBs detected by global proteomics. G) Heat map of LC-MS/MS metabolomics showing raw abundances (colored by row) of up-regulated and down-regulated proteins in the FBs of *FASN1*^*FB-RNAi*^ larvae relative to *Dcg-Gal4* controls. H) Confocal images of control and *FASN1*^*FB-RNAi*^ FBs expressing mitochondrial fluorescent marker *UAS-MitoTimer* to detect changes organelle density and morphology. Scale bar 25 μm, Scale bar (inset) 5 μm.

Since *FASN1*^*FB-RNAi*^ larvae elevate glycogen stores, which upon mobilization release glucose, we queried whether they exhibited enhanced glucose catabolism, beginning with glycolysis. The first step of glycolysis produces glucose-6-phosphate which can be channeled into glycogenesis and the pentose phosphate pathway (PPP), or broken down into pyruvate via a multi-step pathway driven by various enzymes^5,7^ (Fig 3A). Proteomics profiling of *FASN1*^*FB-RNAi*^ larval FBs revealed that enzymes involved in glycolysis including Hex-A, Pgi, Pfk, Gapdh2, Eno, and Pyk (pyruvate kinase) were significantly more abundant compared to controls (Fig 3A,B). Transcript analysis also showed increased mRNA levels for Pyk which produces the end product pyruvate (**SFig 3A**). To confirm that pyruvate itself was elevated in *FASN1*^*FB-RNAi*^ larval FBs, we expressed the fluorescent pyruvate biosensor *UAS-PyronicSF-GFP* in the FB using the *Dcg-Gal4* driver^43^. Indeed, PyronicSF-GFP fluorescence was substantially elevated in *FASN1*^*FB-RNAi*^ FBs compared to controls, indicating pyruvate accumulation (Fig 3C). Next, we queried whether Pyk was required for *Drosophila* development in *FASN1*^*FB-RNAi*^ animals by RNAi-depleting PyK either alone or in combination with FASN1 in the FB. Strikingly, whereas PyK loss alone did not impact *Drosophila* development into flies, FASN1 and PyK dual loss led to ~75% pupal arrest and subsequent death (Fig 3D).

Since sustained glycolysis may also require elevated gluconeogenesis (in addition to glycogenolysis), we also queried whether key gluconeogenic enzymes were necessary for fly development when FASN1 was depleted. We knocked down Pepck (phosphoenolpyruvate carboxykinase), which catalyzes the rate limiting step of gluconeogenesis, and G6P (glucose-6-phosphatase), which converts glucose-6-P to terminal glucose. Indeed, development was not impacted when either was targeted alone, but concurrent loss of Pepck2 or G6P with FASN1 in the FB led to ~87% and ~95% pupal lethality, respectively (Fig 3E). Collectively, this indicates that glycolysis is essential for *FASN1*^*FB-RNAi*^ FB metabolism in animal development.

Pyruvate can be converted into acetyl-CoA and catabolized by the mitochondrial tricarboxylic acid (TCA) cycle and electron transport chain (ETC) to generate ATP energy. During hypoxia, pyruvate is converted to lactate in the cytoplasm and ATP is produced through anaerobic glycolysis only, a less efficient process. This pathway is often upregulated in cancer cells, even in the presence of oxygen, in the so-called Warburg effect^2,44,45^. To determine whether *Drosophila* lacking FB FASN1 relied on anaerobic glycolysis or aerobic respiration for ATP, we first measured mRNA levels of enzymes that catalyze production of lactate and acetyl-CoA. We found that transcripts of lactate dehydrogenase (Ldh), which converts pyruvate to lactate, were not elevated with FASN1 loss (**SFig 3A**). To functionally access if lactate production was necessary to fuel *FASN1*^*FB-RNAi*^ development, we knocked down *Ldh* in the FB either alone or in combination with *FASN1*. Indeed, in both cases *Drosophila* development into flies was not substantially impacted, indicating lactate production was not functionally required for development even in the absence of TG stores (**SFig 3B**). Collectively, this indicates that *FASN1*^*FB-RNAi*^ larvae accumulate glycolytic enzymes and may functionally require glucose catabolism for development. Pyruvate levels are elevated with FASN1 depletion, but lactate metabolism is not necessary.

### Proteomic and metabolic profiling indicate FASN1-KD larvae functionally down-regulate mitochondrial TCA, OXPHOS, and FAO metabolism

Acetyl-CoA derived from pyruvate can be partitioned to *de novo* lipogenesis or to the TCA cycle, which generates metabolic intermediates for energy^4,5^. However, in the absence of the key fatty acid synthesis enzyme *FASN1*, acetyl-CoA should be channeled into the TCA cycle, resulting in increased TCA activity. Surprisingly, proteomics profiling indicated that most TCA enzymes were slightly *decreased* in abundance in *FASN1*^*FB-RNAi*^ larval FBs compared to controls (Fig 3F). In contrast, ATP citrate lyase (ATPCL), which converts the first TCA cycle metabolite, citrate, into acetyl-CoA and oxaloacetate, was markedly elevated in *FASN1*^*FB-RNAi*^ FBs (Fig 3F). ATPCL represents a cataplerosis enzyme enabling TCA intermediates to leave the cycle^43^ (Fig 3A). In line with this, proteomics indicated decreased levels of mitochondrial ETC proteins involved in oxidative phosphorylation (OXPHOS) in *FASN1*^*FB-RNAi*^ FBs compared to controls, suggesting mitochondrial ETC metabolism may be dampened in FASN1-depleted FBs (**SFig 3C**).

These observations suggested that, in contrast to glycolysis, TCA and ETC pathways were not elevated in *FASN1*^*FB-RNAi*^ FBs. We tested how depletion of enzymes that channel carbon into the TCA cycle impacted *Drosophila* development. First, we RNAi depleted MPC1 that transports pyruvate from the cytosol to mitochondria (the site of TCA metabolism), as well as Pdha, which generates acetyl-CoA, the entry substrate of the TCA cycle. FB-specific MPC1 depletion in *FASN1*^*FB-RNAi*^ did result in ~50% reduction in fly development, but this appeared less severe than perturbing glycolysis (**SFig 3D**). Furthermore, FB-specific depletion of Pdha in combination with *FASN1*^*FB-RNAi*^ only mildly impacted larval development into flies (**SFig 3E**). We also monitored TCA cycle intermediate metabolites using LC-MS/MS metabolomics. TCA metabolites including malate, fumarate, citrate/isocitrate, succinate, and alpha-ketoglutarate were elevated in FASN1-depleted FBs, suggesting some alteration of the TCA cycle (Fig 3G). Collectively, this supports a model where *FASN1*^*FB-RNAi*^animals primarily rely on glycolysis to fuel their developmental life cycle, and may be less reliant on pyruvate oxidation.

Since tissues also generate energy through fatty acid oxidation (FAO)^46,47^, we monitored mitochondrial and peroxisomal FAO enzyme levels by proteomics. Mitochondrial and peroxisomal FAO enzymes were both significantly decreased in *FASN1*^*FB-RNAi*^FBs, suggesting FAO may be blunted with FASN1 loss (**SFig 3F**). To evaluate whether mitochondrial FAO was suppressed in FASN1-depleted FBs, we monitored levels of acylcarnitines and other bioenergetic metabolites via LC-MS/MS metabolomics. Indeed, *FASN1*^*FB-RNAi*^FBs exhibited reduced levels of several long-chain acylcarnitines compared to control FBs, suggesting potentially decreased lipid channeling into FAO^48^ (Fig 3G). In contrast, *FASN1*^*FB-RNAi*^FBs displayed elevated intermediate and end metabolites in glycolysis such as fructose-6-phosphate, dihydroxyacetone phosphate, and acetyl-CoA, consistent with elevated glycolysis. We also noted elevated metabolites of the pentose phosphate pathway (PPP), likely due to elevated carbohydrate metabolism in FASN1-depleted FBs (Fig 3A,G).

Finally, we interrogated mitochondrial morphology in L3 larval FBs by using fluorescence confocal microscopy. Whereas control FBs displayed numerous tubular mitochondria throughout the cytoplasm, *FASN1*^*FB-RNAi*^FBs displayed fewer and fragmented mitochondria (Fig 3H). As fragmented mitochondria can be correlated with reduced mitochondria metabolic activity, this supports a model where loss of FASN1 in the FB reduces mitochondrial energetics^49^. Collectively this indicates that *FASN1*^*FB-RNAi*^larvalFBs require glycolysis but may remodel or blunt mitochondrial metabolism during development.

### FASN1^FB-RNAi^ flies are short-lived, starvation sensitive, and require dietary sugar and trehalose for development

**Figure 4:**
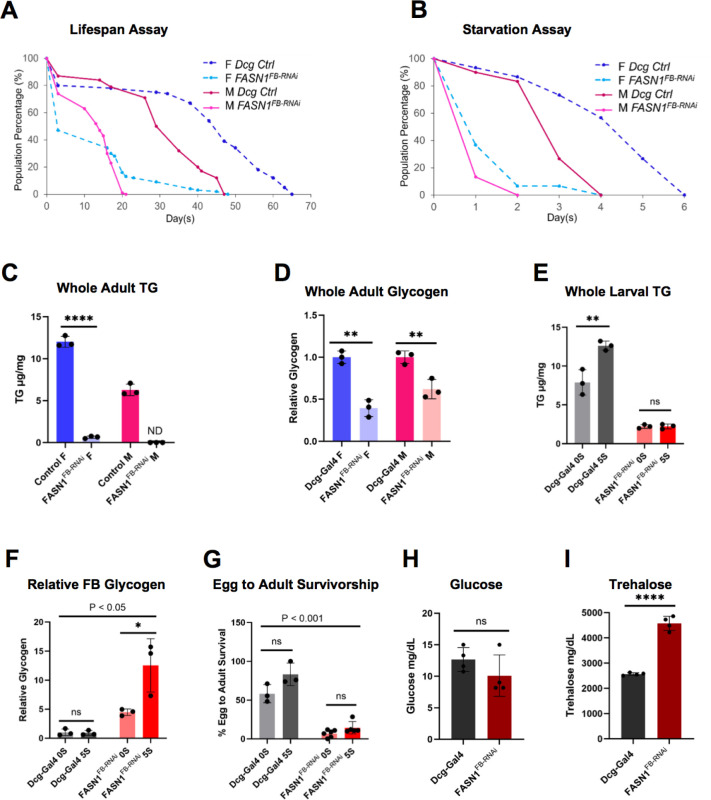
*FASN1*^*FB-RNAi*^ development to adults requires diet-dependent glycogen storage and trehalose production in the FB A) Longevity assay performed for control (*Dcg-Gal4*) and *FASN1*^*FB-RNAi*^ adult females and males (n = 100, each). B) Starvation assay using 7–10 days old control and *FASN1*^*FB-RNAi*^ female and male flies (n = 30, each). C) TG measured in control and *FASN1*^*FB-RNAi*^ male and female flies. D) Relative glycogen measured in control and *FASN1*^*FB-RNAi*^ male and female flies. E) TG measured in control and *FASN1*^*FB-RNAi*^ larvae reared on 0% and 5%sucrose (basic media) diet. F) Glycogen measured in control and *FASN1*^*FB-RNAi*^ larvae reared on 0% and 5% sucrose (basic media) diet. G) Percentage of control and *FASN1*^*FB-RNAi*^ eggs developing to adults on 0% and 5% sucrose diet. H) Control and *FASN1*^*FB-RNAi*^ larval hemolymph glucose levels (mg/DL). I) Control and *FASN1*^*FB-RNAi*^ larval hemolymph trehalose levels (mg/DL).

Given that FB depletion of FASN1 led to metabolic remodeling that reduced mitochondrial bioenergetics and elevated glycogen storage and glycolysis, we next investigated how this influenced organismal lifespan and stress responses. We first examined how *FASN1*^*FB-RNAi*^ male and female adult lifespans compared to controls, discovering that *FASN1*^*FB-RNAi*^ flies of both sexes displayed significantly shortened median lifespan on standard food (Fig 4A). We also found *FASN1*^*FB-RNAi*^ flies were starvation sensitive when maintained on water but no food source, and controls survived approximately twice as long under these fasting conditions (Fig 4B). This was consistent with biochemical analysis indicating that *FASN1*^*FB-RNAi*^ adults displayed significantly lower TG and glycogen levels than control animals, and again indicates that the elevated glycogen in FASN1-depleted larvae is consumed during metamorphosis (Fig 4C,D).

Since *FASN1*^*FB-RNAi*^ larvae exhibited elevated glycolysis and glycogen stores, we monitored whether they required dietary sugar for development. We created a minimal defined food by mixing agar and yeast powder supplemented with varying amounts of sucrose (0% = no sugar or 0S, 5% = standard sugar or 5S). We measured TG and glycogen of control and *FASN1*^*FB-RNAi*^ larvae cultured on 0S and 5S food. In control animals, TG stores were boosted with the consumption of 5S versus 0S, but FB glycogen remained unchanged between larvae eating these diets (Fig 4E,F). Conversely, in FASN1-deficient larvae TG levels were similar between larvae cultured on 0S or 5S food (and were ~80% lower than controls on 5S food), consistent with a blockage of TG synthesis even in the presence of additional dietary sugars. Furthermore, FBs of 0S-fed *FASN1*^*FB-RNAi*^ larvae stored ~65% less glycogen than those fed 5S food, indicating their glycogen stores were largely maintained by dietary sugars (Fig 4F). Though FB glycogen in *FASN1*^*FB-RNAi*^ larvae fed 0S food were ~4-fold elevated relative to controls on 0S, successful development of *FASN1*^*FB-RNAi*^ larvae to adults was significantly diminished on both 0S or 5S food compared to controls (Fig 4G). Collectively, this indicates that *FASN1*^*FB-RNAi*^ *Drosophila* rely on dietary sugars to store the nutrient reserves for development into flies, and in the absence of sufficient dietary sugar fail to store sufficient nutrients to complete metamorphosis.

Since *Drosophila* energy homeostasis is influenced by sugars in the animal hemolymph, we also examined how the disruption of dietary carbohydrate-to-fat conversion and enhanced glycogen storage impacted circulating glucose and trehalose. We measured these in control and *FASN1*^*FB-RNAi*^ pre-wandering L3 larvae. Notably, glucose levels were not altered but trehalose levels were substantially elevated in *FASN1*^*FB-RNAi*^ larvae, suggesting that glycogen in *FASN1*^*FB-RNAi*^ FBs increased trehalose concentrations in circulation to aid animal development (Fig 4H,I). To test this, we RNAi depleted the trehalose synthesis enzyme Tps1 in the FBs of control and *FASN1*^*FB-RNAi*^ larvae. Strikingly, whereas Tps1 loss alone in the FB did not impact development, dual depletion of Tps1 and FASN1 led to significant developmental arrest (**SFig 4A**). Collectively, this indicates that metabolic re-wiring in *FASN1*^*FB-RNAi*^ *Drosophila* creates a dietary sugar dependency, and results in shortened lifespan and starvation sensitivity. It also reveals that *FASN1*^*FB-RNAi*^ larvae require glycogen reserves and trehalose to support development.

### FB-specific FASN1 loss causes female infertility and reproductive arrest

**Figure 5:**
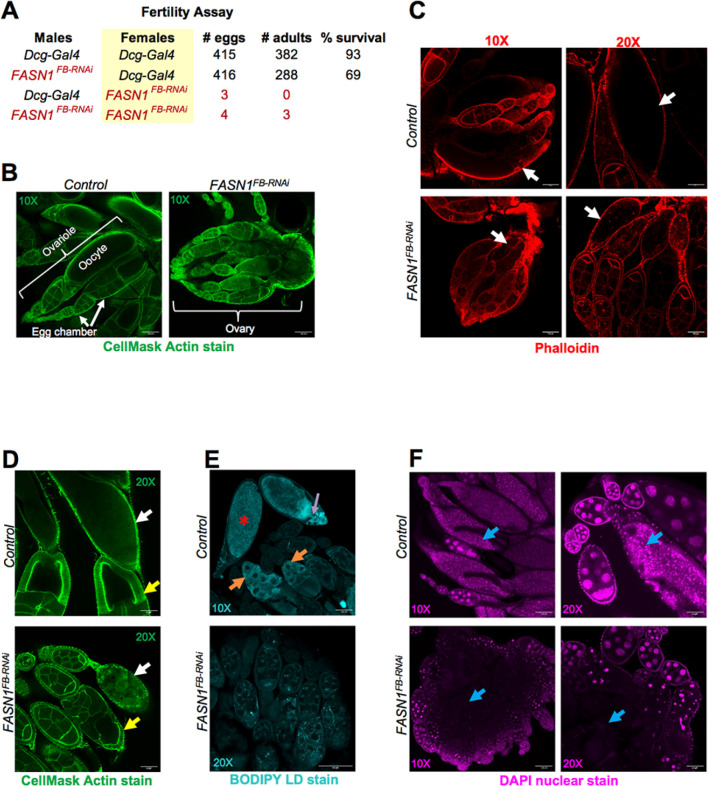
*FASN1*^*FB-RNAi*^ females display arrested oogenesis and are infertile A) Fertility assay performed to test viability of eggs produced by control (*Dcg-Gal4*) or *FASN1*^*FB-RNAi*^ females mated with either control or *FASN1*^*FB-RNAi*^ males. B) Confocal images of ovaries from 7–10-day old control and *FASN1*^*FB-RNAi*^ adult females stained with CellMask Actin stain. Entire *FASN1*^*FB-RNAi*^ ovary (right) is visible at 10X magnification. Parts of the control ovary are labelled (left). Scale bar 100 μm. C) Confocal images of phalloidin-stained ovaries from control and *FASN1*^*FB-RNAi*^ females. White arrows point to mature oocyte in control ovary and arrested oocyte in *FASN1*^*FB-RNAi*^. Scale bar (20X) 100 μm, Scale bar (20X) 50 μm. D) Confocal images of ovaries from control and *FASN1*^*FB-RNAi*^ females stained with CellMask Actin stain. Yellow arrows point to follicle cells of stage 9/10 egg chambers. White arrows point to oocyte; oocyte maturation fails in *FASN1*^*FB-RNAi*^ ovaries. Scale bar 50 μm. E) Confocal images of LDs in control and *FASN1*^*FB-RNAi*^ ovaries stained with BODIPY. Orange arrow in control ovary indicates LDs accumulated in nurse cells and purple arrow indicates ring canal where movement of LDs from nurse cells to the developing oocyte is occurring. There also appear to be plentiful LDs in the mature oocyte*. LDs are sparse in *FASN1*^*FB-RNAi*^ egg chambers. Scale bar (20X) 100 μm, Scale bar (20X) 50 μm. F) Confocal images of ovaries from control and *FASN1*^*FB-RNAi*^ females stained with nuclear stain DAPI. Blue arrow in control ovary point to distinct nuclei in mature oocytes, absent in *FASN1*^*FB-RNAi*^ ovary. Scale bar (20X) 100 μm, Scale bar (20X) 50 μm.

As animal nutrient reserves are tightly linked with reproductive success^50,51^, we next examined how loss of FASN1 in the FB impacted fecundity by monitoring whether *FASN1*^*FB-RNAi*^ female and male flies could successfully generate progeny. Indeed, eggs deposited by control (*Dcg-Gal4*) females mated with *FASN1*^*FB-RNAi*^ males showed slightly decreased viability relative to those from *Dcg-Gal4* fathers, suggesting loss of FASN1 in the FB of males mildly perturbed their fecundity (Fig 5A). Strikingly, *FASN1*^*FB-RNAi*^ females mated with either control or *FASN1*^*FB-RNAi*^ males failed to lay eggs, indicating *FASN1*^*FB-RNAi*^ females exhibited substantial fertility defects (Fig 5A).

The adult *Drosophila* female reproductive system consists of two ovaries, each with 20–30 ovarioles. An ovariole is organized into a procession of egg chambers (Fig 5B). Early egg chambers are made of 16 sister cells enclosed by a monolayer of somatic follicle cells. A single nurse cell is fated to become an oocyte, while the remaining 15 nurse cells supply the growing oocyte with nutrients, proteins, and RNA. Oocyte maturation within the egg chamber occurs in 14 morphologically distinct stages^52,53^. Dissection of the internal reproductive organs of *FASN1*^*FB-RNAi*^ females revealed shrunken and underdeveloped ovaries which did not produce mature oocytes (Fig 5B,C, **white arrows**). Upon close examination, *FASN1*^*FB-RNAi*^ egg chambers appeared to arrest at stage 9–10, when normally the follicle cells migrate to surround the oocyte (Fig 5D, follicle cells denoted by **yellow arrows**). This also represents a critical oogenesis checkpoint when extracellular diacylglycerol (DG), sourced primarily from the FB, are absorbed by nurse cells via a lipophorin receptor (LpR2) dependent pathway. DG is acylated to TG and assimilated in LDs, which accumulate in nurse cells and are transported through connecting ring canals to the developing oocyte to drive its maturation and later fuel embryogenesis^52–55^. In contrast to controls, *FASN1*^*FB-RNAi*^ ovaries were largely devoid of LDs (Fig 5E, **orange arrows**). This suggests they may have failed to clear this key metabolic checkpoint, resulting in degeneration of the egg chambers as no discernable cells with nuclear DAPI staining were visible in the interior of *FASN1*^*FB-RNAi*^ ovaries (Fig 5F, **blue arrows**) Collectively, we conclude that FB-specific FASN1 loss leads to female *Drosophila* infertility due to defects in lipid delivery to ovaries for oogenesis.

### FASN1-depleted FBs exhibit elevated oxidative stress signatures and rely on antioxidants for development

Our metabolic and proteomic profiling revealed that in addition to increased glycolysis, *FASN1*^*FB-RNAi*^ FBs elevated pentose phosphate pathway (PPP) metabolism, which generates the redox metabolite nicotinamide adenine dinucleotide (NADPH) as a bi-product. NADPH is an electron donor and key metabolite in redox homeostasis^5,23^. Importantly, NADPH is required for FASN-dependent *de novo* lipogenesis, and inhibiting FASN activity can elevate cellular NADPH pools which perturb redox balance and drive oxidative stress through NAPDH Oxidase (NOX)-mediated reactive oxygen species (ROS) generation^47,56^. For example, cancer cells typically elevate FASN-dependent lipogenesis to support anabolic metabolism and cell growth. Pharmacological FASN inhibition is an anti-cancer strategy that can drive NADPH accumulation in cancer cells, promoting ROS signaling and cell death via NOX activity^4,57^.

**Figure 6:**
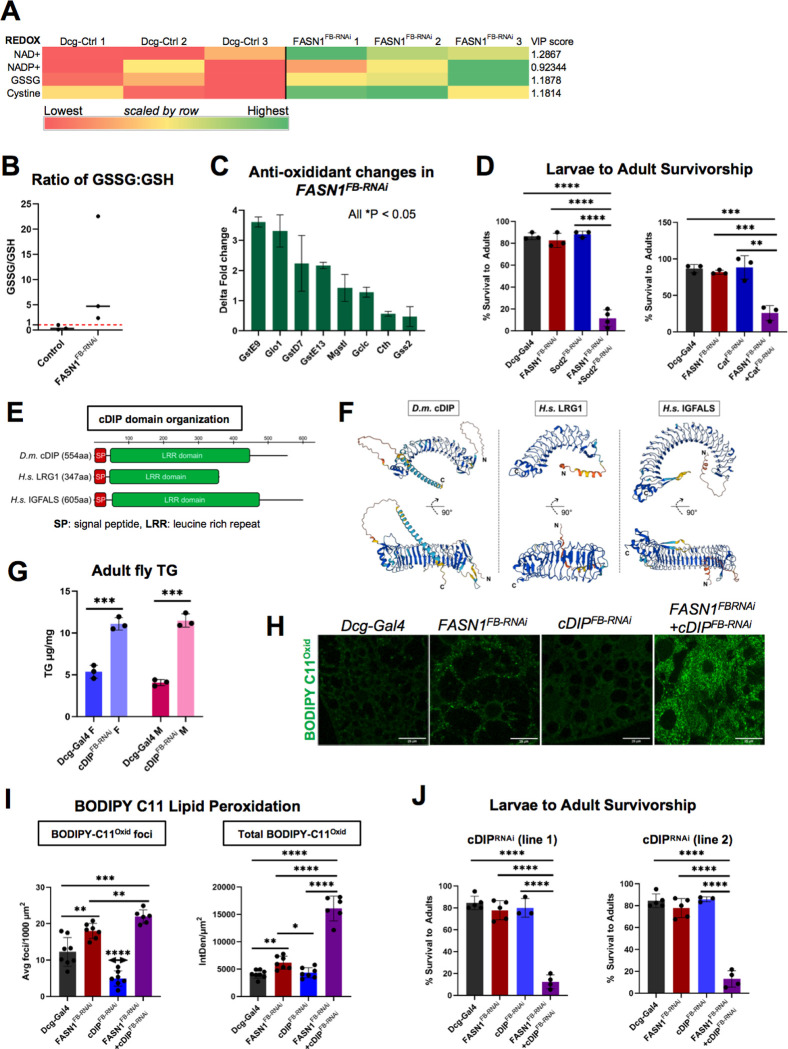
*FASN1*^*FB-RNAi*^ animals suffer from oxidative stress and require antioxidants to complete development A) Heat map of oxidation products of metabolites maintaining redox balance in the FBs of *FASN1*^*FB-RNAi*^ larvae relative to Dcg-Gal4 controls (raw abundances). B) Ratios of glutathione disulfide (oxidized) to glutathione (reduced) in control and *FASN1*^*FB-RNAi*^ FBs. C) Relative changes (delta fold) in proteins involved in glutathione metabolism in *FASN1*^*FB-RNAi*^ FBs relative to control. D) Percentage of control and *FASN1*^*FB-RNAi*^ larvae, as well as those deficient of antioxidants *Sod2* or catalase, alone or also without FASN1, successfully developing into adult flies. E) Domain architecture of *Drosophila melanogaster* (*D.m*.) cDIP, *Homo sapiens* (*H.s*.) LRG1, and *H.s*. IGFALS. F) AlphaFold2 structural predictions of *D.m*. cDIP, *H.s.* LRG1, and *H.s*. IGFALS. Confidence score is colored blue-to-red (high-to-low) according to AlphaFold2. G) Triglyceride (TG) levels for male (M) and female (F) adult control (*Dcg-Gal4*) and *Dcg-Gal4>cDIP*^*RNAi*^ flies. H) Confocal images of BODIPY-C11 stained larval FBs from control (*Dcg-Gal4*), or *Dcg-Gal4*-driven *cDIP*^*RNAi*^, *FASN1*^*RNAi*^, or *FASN1*^*RNAi*^+ *cDIP*^*RNAi*^ dual depletion. I) Quantification of BODIPY-C11^Oxid^ puncta and signal intensity in control, *FASN1*^*FB-RNAi*^, *cDIP*^*FB-RNAi*^, and *FASN1*^*FB-RNAi*^+ *cDIP*^*FB-RNAi*^ FBs. Also, quantification of BODIPY-C11^Oxid^ foci (#foci/100 square microns) and total signal (IntDen/square micron) for experiments in Panel H. J) Percentage of L3 larvae (control, *FASN1*^*FB-RNAi*^, *cDIP*^*FB-RNAi*^, *FASN1*^*FB-RNAi*^+ *cDIP*^*FB-RNAi*^) developing to adult flies. Two independent *UAS-cDIP*^*RNAi*^ lines were tested.

Given that FASN1 FB-depleted animals displayed shortened lifespans, we investigated whether they exhibited signatures of altered redox homeostasis. Indeed, metabolomic profiling indicated *FASN1*^*FB-RNAi*^FBs contained elevated concentrations of oxidized species of several redox related metabolites including NADP+, NAD+, cystine, and glutathione disulfide (GSSG), the oxidized form of glutathione (GSH)^24,58,59^ (Fig 6A). In line with this, GSSG:GSH ratios were significantly higher in *FASN1*^*FB-RNAi*^larvae compared to controls (Fig 6B). Proteomics also indicated *FASN1*^*FB-RNAi*^FBs had elevated glutathione S-transferases (GST) and related enzymes, suggesting FASN1 loss elevated oxidative toxins that required GST activity (Fig 6C).

Given that *FASN1*^*FB-RNAi*^ larvae displayed signatures of oxidative stress, we interrogated whether depletion of major cellular antioxidants negatively impacted larva-to-adult development. We targeted three major antioxidants in the FB: superoxide dismutase 1 (SOD1), SOD2, and catalase. We RNAi depleted these factors either alone or in combination with FASN1 specifically in the FB using the *Dcg-Gal4* driver. Notably, whereas RNAi-depletion of SOD1 or SOD2 alone had no impact on fly development, their depletion together with FASN1 led to a ~ 29% and ~70% reduction in fly eclosion, respectively (Fig 6D, **SFig 6A**). Similarly, RNAi depletion of peroxisome-localized catalase did not affect *Drosophila* development, but its co-depletion with FASN1 led to ~75% reduction in adult development (Fig 6D). In line with this, *FASN1*^*FB-RNAi*^ larval FBs displayed significantly more peroxisomes compared to controls, suggesting peroxisomes may be elevated in FASN1-depleted FBs to support homeostasis (**SFig 6B**). In support of this, RNAi depletion of peroxisomal biogenesis factor Pex5 in conjunction with FASN1 reduced larva-to-fly development (**SFig 6C**). Collectively, this indicates that FASN1 depleted FBs display signatures of defective redox homeostasis and functionally require antioxidant enzymes and peroxisomes for developmental integrity.

### cDIP is required for development of FASN1-depleted larvae and influences FB energy and redox homeostasis

Given that *FASN1*^*FB-RNAi*^ *Drosophila* are fat-deficient, reliant on carbohydrate metabolism for development, and displayed signatures of oxidative stress, we reasoned they may elevate the expression of signaling molecules that influenced energy homeostasis of the FB. To potentially uncover such new signaling molecules, we examined our FB proteomics for uncharacterized proteins with signatures of signaling functions (Fig 2A). We noted that among the most up-regulated proteins (~70-fold) in the *FASN1*^*FB-RNAi*^ proteomics was Common Dpr-interacting Protein (cDIP)/CG10824, a poorly characterized leucine rich-repeat (LRR) protein. Panther and Phylome protein phylogeny databases indicate cDIP has low sequence homology to several LRR proteins that function as adipokines or insulin-like growth factor binding proteins (IGFBPs), secreted factors that fine-tune endocrine signaling by directly binding insulin or IGF-like peptides to regulate their serum lifetime or receptor bioavailability^60^. For example, cDIP displays limited homology (27% identical, 41% similar amino acids) to vertebrate IGF-binding protein acid-labile subunit (IGFALS), a secreted factor that binds circulating insulin growth factor-1 (IGF-1) to influence growth and energy storage^61,62^. cDIP also displays some similarity (33% identical, 23% similar amino acids) to Leucine-rich alpha-2-glycoprotein 1 (LRG1) a secreted obesity-linked cytokine that regulates insulin signaling^63,64^. Indeed, cDIP’s domain architecture closely resembles these factors with a predicted N-terminal signal peptide and LRR domain (Fig 6E). Furthermore, AlphaFold2 structural predictions suggest structural similarities between cDIP, LRG1, and IGFALS, implying potential functional similarities in energy signaling (Fig 6F). Based on this, and since *Drosophila* feature a simplified insulin/IGF-like signaling (IIS) system that governs energy storage, we hypothesized cDIP may function in some aspect of energy homeostasis.

To probe into the functional relevance of cDIP, we RNAi-depleted cDIP in the FB (*Dcg-Gal4>cDIP*^*RNAi*^), and monitored whole body TG levels. cDIP loss did not alter TG in larvae but led to fly obesity with a striking >2-fold TG increase in adult male and female flies compared to controls (Fig 6G). Since obesity often correlates with elevated oxidative stress^65^, we also queried whether cDIP loss impacted FB redox homeostasis in the presence and absence of FASN1. We stained larval FBs with the lipid peroxidation biosensor BODIPY-C11. Reduced BODIPY-C11 exhibits a red fluorescence emission of ~590nm but shifts to green (~510nm) emission when oxidized^66^. In control larval FBs, we noted red (reduced) BODIPY-C11 (BODIPY-C11^Red^) which was incorporated in LDs (Fig 6D). This is consistent with previous studies indicating that LDs serve as lipo-protective depots that reduce lipid oxidation^28^. Control FBs exhibited a diffuse dim green (oxidized) BODIPY-C11 (BODIPY-C11^Oxid^), presumably decorating cellular membranes, and a few green foci were observed (Fig 6H). In contrast, *FASN1*^*FB-RNAi*^FBs displayed significantly brighter BODIPY-C11^Oxid^ signal and more foci, consistent with oxidative stress (Fig 6H,I). As expected, there was significantly less BODIPY-C11^Red^ associated with LDs in *FASN1*^*FB-RNAi*^ tissues since they were largely depleted of fat, consistent with the concept that loss of LDs promotes lipid peroxidation (**SFig 6D**).While loss of cDIP alone did not elevate BODIPY-C11^Oxid^ intensity in FB cells, its dual depletion with FASN1 led to significantly elevated BODIPY-C11^Oxid^ signal and foci, suggesting cDIP loss perturbed some aspect of redox homeostasis (Fig 6H,I).

Given that cDIP appeared to influence energy storage and redox homeostasis in the FB, we queried whether Drosophila required cDIP for development. Strikingly, for two independent cDIP^RNAi^ lines we noted that whereas FB-specific depletion of cDIP alone did not impact *Drosophila* development, its joint loss with FASN1 in the FB resulted in a near complete loss of fly eclosion, and animals died as pupae (Fig 6J). Collectively, this indicates that cDIP influences some aspect of energy homeostasis and is essential for *Drosophila* development in the absence of fat stores. We hypothesize that cDIP is secreted from FB cells in response to depletion of fat stores and facilitates metabolic remodeling by blunting energy signaling to the FB, possibly by regulating IIS activity. As such, we propose cDIP loss may perturb the ability of FASN1-depleted larvae to rebalance their metabolism towards glycolysis and catabolic metabolism. Future studies will dissect how cDIP modulates energy and redox homeostasis.

## Discussion

Energy balance is largely reliant on glucose, which drives glycolysis, the TCA cycle, and OXPHOS, as well as adipose tissue metabolism^3,4,67^. Dietary sugars stimulate expression of *de novo* lipogenic enzymes including acetyl CoA carboxylase (ACC) and FASN that convert acetyl CoA derived from pyruvate to fatty acids that are esterified to TG and assimilated into LDs^5,18^. In *Drosophila*, the FB which is the central lipid depot, performs liver-like functions and stores both TG and glycogen^18,67^. We capitalized on this TG:glycogen dual utilization to identify factors mediating energy balance and adaptation in animal development. We find that FB-specific loss of FASN1 leads to a near complete loss of TG, and rebalancing to carbohydrate-oriented metabolism. FASN1-depleted larvae maintain normal FB tissue size and complete their developmental life cycle via elevated glycogen storage and glycolysis, but manifest adult female infertility and shortened lifespans. FASN1-depleted FBs also display signatures of oxidative stress and lipid peroxidation. We also identify cDIP, a putative cytokine-like factor with homology to secreted IIS factors IGFALS and LRG1, as critical to FB energy homeostasis when FASN1 is lost. Our work suggests cDIP may act as a ‘sensor’ of fat depletion in the FB, and coordinator of metabolic remodeling that rewires FB metabolism away from nutrient storage and towards catabolic metabolism.

*Drosophila* must reach critical weight to achieve pupation and metamorphosis, and the biomass for this is generally thought to come from FB lipids^24–26^. We find *FASN1*^*FB-RNAi*^ larvae lack lipid stores yet grow to full size and pupate without developmental delay. We hypothesize that the ~20-fold concentrated glycogen deposits in *FASN1*^*FB-RNAi*^ FBs may increase body mass by elevating FB cell water content. Glycogen absorbs 3–5 times its weight in water which may help compensate for lipid loss and help *FASN1*^*FB-RNAi*^ larvae to reach critical weight^68^. Additionally, our data indicate that glycogen reserves, previously deemed dispensable for metamorphosis^6,8^, are essential for *FASN1*^*FB-RNAi*^ animals during pupal to pharate adult transition, while control animals rely mostly on TG. This metabolic rewiring reveals a previously unappreciated versatility to *Drosophila* development.

Transcriptomics and proteomics identified tobi, an alpha-glucosidase, as essential to bypass TG loss in FB tissue. Similarly, GO term analysis indicated significant reprogramming of glucose metabolism, whereby energy was channeled back into carbohydrates via glycogenesis and possibly gluconeogenesis, and metamorphosis was primarily fueled by aerobic glycolysis (but without lactate production, as in the Warburg effect). In line with this, loss of tobi, essential for glycogenolysis, or enzymes involved in gluconeogenesis, or glycolysis is fatal in *FASN1*^*FB-RNAi*^ animals, but not in controls. It is known that FB glycogen, generally labelled a ‘metabolic safeguard’, can be mobilized to replenish trehalose in circulation during nutrient shortages^69^. Trehalose may be directly synthesized from glucose by trehalose-6-phosphate synthase (Tps1) exclusively in the FB^70^. Interestingly, recent studies show that 90% of hemolymph trehalose is utilized in early metamorphosis and *Tps1* null mutants do not complete metamorphosis^32,33^. We found that while FB-specific *Tps1-RNAi* larvae developed into adults, loss of *Tps1* and *FASN1* together resulted in a high degree of pupal lethality, suggesting that both FB glycogen and hemolymph trehalose support fly development in the absence of TG. Indeed, we detected higher amounts trehalose in *FASN1*^*FB-RNAi*^ larval hemolymph, probably owing to enhanced glycogen breakdown and gluconeogenesis in these animals.

Fat depleted *Drosophila* maintain their developmental integrity through rebalancing fat:carbohydrate metabolism, but does this expose new vulnerabilities? While glycogen metabolism facilitated *Drosophila* development, fertility was adversely affected in *FASN1*^*FB-RNAi*^ females. *FASN1*^*FB-RNA*^ ovaries appeared morphologically small and underdeveloped. This may be a consequence of a phenomenon called “organ sparing”. When energy supply is limited, animals preferentially support development of certain organs at the cost of others^51^. In *FASN1*^*FB-RNAi*^ female flies, we noted that egg chamber oocyte selection from nurse cells, and wrapping by follicle cells, both occurred improperly, stalling oocyte maturation around stage 9–10 of oogenesis. Since maternal TG deposition is a critical checkpoint for oocyte maturation and embryo survival, failure of oogenesis in *FASN1*^*FB-RNAi*^ females likely results from low level of LDs in nurse cells. TG stored in LDs of nurse cells is synthesized from DAG, 80% of which is exogenously resourced from free-floating cells of the dissociated larval FB that persist in the newly hatched fly^28,55^. We reason that lipophorin-mediated DAG delivery from *FASN1*^*FB-RNAi*^ lipid-deficient fat cells to the ovaries was severely limited, resulting in organ developmental arrest. In line with this, mid-oogenesis arrest and subsequent apoptosis of egg chambers due to low lipid content has been reported by other studies^71,72^.

Though *FASN1*^*FB-RNAi*^ larvae were able to complete metamorphosis and become adults, flies harbored signatures of metabolic and redox stress. Adult flies exhibited barely detectable TG stores and half the glycogen reserves of controls; unsurprisingly this rendered them starvation sensitive. *FASN1*^*FB-RNAi*^ male and females displayed shorter median lifespans relative to controls. We hypothesize this may be, in part, due to elevated ROS production in these animals, as ROS has been linked to accelerated aging^73^. Metabolomic profiling revealed significant elevations in the ratio of oxidized glutathione GSSG:GSH, and factors involved GSH redox cycling in *FASN1*^*FB-RNAi*^ FBs. Furthermore, *FASN1*^*FB-RNAi*^ animals required SOD and catalase antioxidants for development. Of note, cDIP depletion in the FB elevated not only nutrient storage but also redox stress as indicated by BODIPY-C11 staining. The source of ROS production is unclear but may be linked to elevated oxidative pathways coupled to energy storage in the FB. Additionally, FASN1 depletion may elevate NADPH, a substrate of de novo lipogenesis and bi-product of the pentose phosphate pathway, which is also upregulated in *FASN1*^*FB-RNAi*^ animals. NADPH accumulation can drive oxidative stress via NAPDH-dependent oxidase (NOX) ROS production. Alternatively, elevated metabolism in the FASN1-depleted background may drive ROS signaling from several pathways. Further studies will dissect how FASN1 loss elevates oxidative stress, and how cDIP both coordinates energy metabolism and redox homeostasis at the FB.

## Materials And Methods

### Fly cultures and strains

All *Drosophila* animal research was performed in accordance with NIH recommended policies. *Drosophila* L3 late feeding/pre-wandering larvae were used for fat tissue microscopy and biochemical assays, unless otherwise stated. For lifespan and starvation assays, adult male and female flies were used. Flies were reared and maintained on standard fly food containing cornmeal, yeast, molasses (~5% sugars), and agar. Basic media for chronic sugar feeding experiments was prepared with only yeast (10%) and agar (1.5%), supplemented with varying concentrations of sucrose (0% or 5%). The *Dcg-Gal4* fat body (FB)-specific driver^38^ was provided by Jonathan M. Graff (former UTSW). *UAS-FASN1*^*RNAi*^and all other *TRiP RNAi* lines, *PyronicSF-GFP*, protein/organelle marker strains and other transgenic stocks used in this study were obtained from the Bloomington Stock Center (Bloomington, IN). All Gal4/UAS crosses were cultured at 25°C to maintain uniform transgene expression.

### Larval fat body/ gut dissection and staining

Late feeding larvae were gently removed from inside the food media using a paintbrush and rinsed in water to remove food particles. Larval fat bodies (FBs) or guts were dissected in PBS using Dumont #5 forceps (Electron Microscopy Sciences). All tissues were fixed in 4% paraformaldehyde (PFA) for 20 min at room temperature and rinsed briefly in PBS prior to staining. Lipid droplets in FBs were stained by incubating FBs in 100 μM monodansylpentane (MDH) LD stain (Abgent) for 20 min at room temperature, in the dark. LDs in guts were stained with 1 μM Nile Red for 30 min at room temperature, in the dark. Next, FBs and guts were rinsed in PBS and mounted on slides in SlowFade Gold antifade reagent with nuclear DAPI (Invitrogen) for subsequent imaging.

### Female ovary dissection and staining

Ovaries from 7 to 10-day old adult females were dissected in PBS, fixed in 4% PFA for 20 min at room temperature. Following a brief wash in PBS, ovaries were incubated in CellMask Actin stain (1:1000) or Phalloidin (1:400 in PBS-T) for staining of F-actin structures, according to manufacturer’s instructions (Invitrogen). LDs in ovaries were stained with BODIPY 493/503 C12 (1:500) for 30 mins in the dark. Excess stain from ovaries was removed by washing in PBS, then mounted in SlowFade Gold antifade reagent containing DAPI (Invitrogen) for imaging.

### Confocal fluorescence microscopy

Prepared slides were imaged using Zeiss LSM880 inverted laser scanning confocal microscope. Tissues stained with organelle dyes (LD: MDH/ BODIPY C12/Nile Red; F-actin: phalloidin) and those expressing transgenic fluorescent-tagged proteins (e.g., UAS-GFP; ER marker *UAS-Sec61B-TdT*; PM marker *UAS-mcd8-GFP*; pyruvate sensor *PyronicSF-GFP*; mitochondrial marker *UAS-MitoTimer*) were imaged using the appropriate channel filter DAPI/GFP/RFP. Most FBs were imaged using a 40X oil immersion objective, and adult female ovaries with 10X or 20X objective.

### Lipid peroxidation assay

FBs dissected from late feeding larvae were incubated in 2 μM of BODIPY 581/591 (Invitrogen, D3861), a fluorescent lipid peroxidation sensor, according to manufacturer’s instructions. Tissues were rinsed in PBS, fixed in 4% PFA for 20 min (in the dark) and washed briefly again in PBS. FBs were mounted in SlowFade Gold antifade reagent, and GFP and RFP channel filters were used to image green (oxidized) and red (reduced) fluorescence in FB cells. GFP and RFP FB intensities were quantified using Fiji ImageJ and oxidized:reduced ratios were calculated.

### Light and other fluorescence microscopy

Whole 3L larvae without or with GFP-expressing (*Dcg-Gal4>UAS-GFP*) FBs were imaged using the Leica S8AP0 stereoscope and EVOS FL Cell Imaging System (ThermoFisher), respectively. Adult male and female flies were also imaged on Leica S8AP0 stereoscope.

### Transmission electron microscopy

FB tissue was removed from *Drosophila* larvae, fixed in 2.5% (v/v) glutaraldehyde in 0.1 M sodium cacodylate buffer paraformaldehyde, and processed in the UT Southwestern Electron Microscopy Core Facility. They were post-fixed in 1% osmium tetroxide and 0.8% K3[Fe(CN6)] in 0.1 M sodium cacodylate buffer for 1 hour at room temperature. Cells were rinsed with water and en bloc stained with 2% aqueous uranyl acetate overnight. Next, they were rinsed in buffer and dehydrated with increasing concentration of ethanol, infiltrated with Embed-812 resin and polymerized in a 60°C oven overnight. Blocks were sectioned with a diamond knife (Diatome) on a Leica Ultracut UCT (7) ultramicrotome (Leica Microsystems) and collected onto copper grids, post stained with 2% aqueous uranyl acetate and lead citrate. Images were acquired on a JOEL 1400 Plus transmission electron microscope using a voltage of 120 kV.

### Quantitative PCR

Whole RNA was extracted from FB tissues using Trizol reagent (Ambion Life Technologies) according to manufacturer’s instructions. RNA was quantified by Denovix spectrophotometer, and 1 μg of RNA was reverse transcribed to cDNA using iScript cDNA Synthesis Kit (BioRad). QPCR was performed with cDNA using the SsoAdvanced Universal SYBR Green Supermix (BioRad) on the BioRad CFX96 Real-Time System; mRNA expression data were normalized to that of the fly housekeeping gene rp49. Primer sequences for all transcripts amplified are available upon request.

### Glucose and Trehalose assays

Feeding larvae washed out from culture vials were collected in 630 μm mesh-fitted baskets (Genesee) and rinsed to get rid of adherent food particles. Larvae were dried and divided onto into piles (10–12 larvae each) on a strip of parafilm. Larvae were bled by tearing the cuticle with Dumont 5 forceps (Electron Microscopy Sciences). Two μl of colorless hemolymph was aspirated from each pile and separately transferred to 96-well plates (Thermo-Scientific) containing 0.1% N-Phenylthiourea (Sigma-Aldrich) in 50 μl PBS. 150 μl of Autokit Glucose reagent (Wako) was added to each well and incubated at room temperature for 20 min before measuring absorbance at 505 nm. Glucose concentration was calculated from a standard curve generated with manufacturer’s glucose standards. For trehalose assays, 8 μl of dilute hemolymph was treated with 5 μl of (diluted 8X) porcine kidney trehalase (Sigma) overnight at 37 °C. Ten μl of treated sample was assayed for trehalose as described for glucose. Trehalose amounts were calculated from standard glucose, as for glucose.

### Glycogen assay

Whole larvae (10 larvae per sample, in triplicate) or dissected FBs (20–40 larvae per sample, in triplicate) were homogenized in 300 μl ice-cold PBS using a pestel or sonication and syringe (29G1/2 needle), respectively. After reserving 20 μl of the homogenate for protein Bradford quantification, the rest of the homogenate was heat inactivated at 70°C for 10 min. Homogenate was then centrifuged at maximum speed at 4°C for 10 min and supernatant was collected in a new tube. In a 96-well plate, 30 μl of each sample was loaded in duplicate rows. Then, 100 μl of Autokit Glucose reagent + amyloglucosidase (2 μl amyloglucosidase {Sigma A1602; 25mg} per 1 ml of Glucose reagent) was added to one row of samples, and 100 μl of Glucose reagent alone (without amyloglucosidase) was added to the duplicate row of samples, and to the glucose standards. The plate was incubated at 37°C for 30 min, after which color intensity was measured using a microplate reader at 505 nm. Free glucose concentration in treated and untreated samples was calculated based on the glucose standard curve. Glycogen concentration (as free glucose) was determined by subtracting glucose in the untreated samples from those treated with amyloglucosidase. Finally, total glycogen was normalized to protein measured by the Bradford assay.

### Periodic Acid-Schiff stain

Glycogen in FB tissues was stained using the Periodic Acid-Schiff (PAS) kit (Sigma). Larval FBs were dissected in 1% BSA in PBS, fixed with 4% paraformaldehyde for 20 min, and washed twice with 1% BSA in PBS. FBs were then incubated in 0.5% period acid solution for 5 min, washed twice with 1% BSA in PBS, then stained with Schiff’s reagent for 15 min, washed again, and mounted on slides. PAS-stained FB tissues were imaged using the Leica DM6B light microscope.

### Thin layer chromatography

Larvae were weighed, then homogenized in 2:1:0.8 of methanol:chloroform:water. Samples were incubated in a 37°C water bath for 1 hour. Chloroform and 1M KCl (1:1) were added to the sample, centrifuged at 3000 rpm for 2 min, and the bottom layer containing lipids was aspirated using a syringe. Lipids were dried using argon gas and resuspended in chloroform (100 μl of chloroform/7mg of fly weight). Extracted lipids alongside serially diluted standard neutral lipids of known concentrations were separated on TLC plates using hexane:diethyl ether:acetic acid solvent (80:20:1, v/v/v). TLC plate was air dried for 10 min, spray stained with 3% copper (II) acetate in 8% phosphoric acid and incubated at 145°C in the oven for 30 min to 1 hour to allow bands to develop for scanning and imaging. Neutral lipid (TAG) band intensity was quantified using Fiji ImageJ software, and lipid concentrations were calculated from the standard curve generated with standard mixture.

### Adult starvation assay

Freshly emerging adult flies were collected over a period of 1 to 3 days and transferred to fresh standard food bottles. Flies were aged for 7 to 10 days, then male and female flies were separately moved to food-free vials (10 flies/vial, in triplicate) containing a cotton plug at the bottom soaked with 2 ml of tap water. Flies were kept at 25°C and vials were examined at least 3X daily to record number of dead flies. Surviving flies were moved to fresh vials with water-soaked plug every 24 hours for optimal hydration and to avoid fungal growth.

### Adult lifespan assay

Flies hatching between 0 to 3 hours were pooled, and 100 males and 100 females were separately placed in fresh standard food vials (20 flies/vial). Vials were maintained at 25°C, and dead flies were scored daily while moving surviving flies to fresh food.

### Adult survivorship assay

Double FB knockdowns of FASN1 and candidate genes were generated by crossing 5 to 7 *Dcg-Gal4>UAS-FASN1-RNAi* males to ~20 females from candidate *UAS-RNAi* line in standard food vials cultured at 25°C. Mated females were allowed to lay eggs for 2 days, then parents were removed to fresh vials. Larvae carrying *Dcg-Gal4* driver + both *UAS-RNAi* transgenes were identified and moved to a new vial for developmental monitoring. Animals successfully completing metamorphosis and hatching into adult flies were scored. This count was compared to adult survivorship of *Dcg-Gal4* control, *Dcg-Gal4>UAS-FASN1-RNAi, Dcg-Gal4>cand UAS-RNAi* animals.

### Fertility assays

All parents used were 7 to 10 days old. 10 virgin females were crossed with 10 males (controls) in fresh food vials streaked with yeast paste and kept overnight at 25°C. The following morning, flies were flipped to new food at 1 hour intervals (3X) to empty females of older eggs/embryos. Flies were then allowed to lay on fresh plates supplemented with a drop of yeast paste for 4–6 hours, following which all parents were removed from bottles and number of eggs laid was counted. The hatching of eggs on the food surface and development of progeny was closely monitored and recorded.

### Sample preparation for proteomic profiling

Fat bodies were extracted from *Drosophila* late feeding larvae in chilled Lysis Buffer + protease inhibitor cocktail. Tissues were homogenized by sonication and supernatant (lysate) was collected after centrifugation for 30 sec at 1000×g at 4°C. Centrifugation was repeated at 16,000×g for 10 min at 4°C, and supernatant transferred to fresh Eppendorf tube. Sample for loading was prepared by mixing protein sample with 2X LDS Sample Buffer and Lysis Buffer, and boiling at 95°C for 5 min. Sample was run on a 10% pre-cast polyacrylamide gel (BioRad) at 100 V for ~25 min until the dye front was past the ladder. Gel was rinsed in MQ-water, then stained with Coomassie BB R250 in 10% acetic acid, 50% methanol, 40% water for 20 min at room temperature with rocking. Subsequently, gel was de-stained for 1 hour in 10% acetic acid, 50% methanol, 40% water with 4 solvent changes. When gel background appeared clear, de-stain was exchanged for water. Protein gel bands were cut on clean glass surface of gel imager with new razor, diced into 1 mm cubes, and moved to sterile Eppendorf tubes on ice for delivery to Proteomics Core Facility at UTSW for protein identification by mass spectrometry.

### Sample preparation for metabolomic profiling

Fat tissues dissected from late feeding larvae were homogenized in ice-cold methanol/water 80:20 (vol/vol) using a sonicator. Lysate was subjected to three freeze-thaw cycles between liquid nitrogen and 37 °C, vortexed vigorously, and then centrifuged at ~20,160×g for 15 min at 4°C. Protein in the supernatant was measured using the Pierce BCA kit, and volume containing 10 μg protein was dried in a SpeedVac. Sample was re-suspended in 100 uL acetonitrile/water 80:20 (vol/vol), vortexed for 1 min, and centrifuged at ~20,160×g for 15 min at 4°C. Metabolite-containing supernatant was transferred to a fresh Eppendorf tube and submitted to the CRI Metabolomics Facility at UTSW for LC-MS metabolite analyses. Metabolites were quantified as described previously ^74,75^.

## Figures and Tables

**Figure 7:**
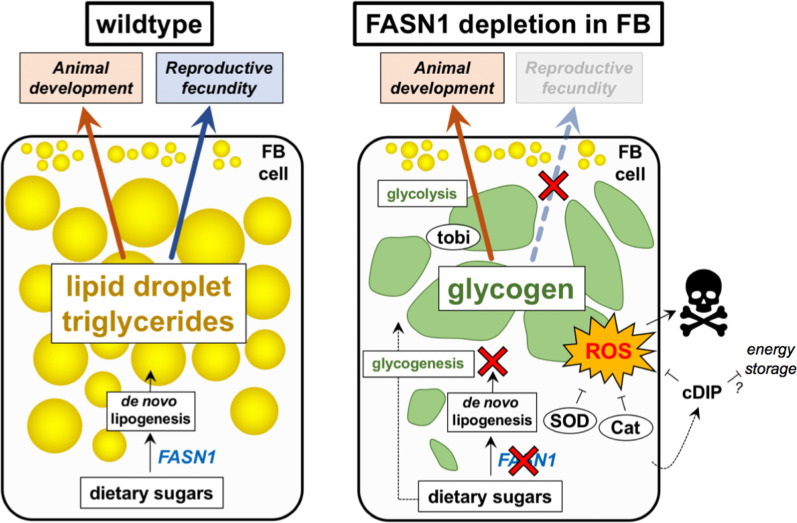
Working model for how triglyceride: carbohydrate cross-talk and metabolic re-wiring occurs in a ‘fat-less’ *Drosophila* to promote growth and development
